# Three-dimensional ultrasound-based radiomics nomogram for the prediction of extrathyroidal extension features in papillary thyroid cancer

**DOI:** 10.3389/fonc.2023.1046951

**Published:** 2023-08-23

**Authors:** Wen-Jie Lu, Lin Mao, Jin Li, Liang-Yan OuYang, Jia-Yao Chen, Shi-Yan Chen, Yun-Yong Lin, Yi-Wen Wu, Shao-Na Chen, Shao-Dong Qiu, Fei Chen

**Affiliations:** Department of Ultrasound, The Second Affiliated Hospital of Guangzhou Medical University, Guangzhou, China

**Keywords:** three-dimensional ultrasound, radiomics, nomogram, extrathyroidal extension (ETE), papillary thyroid cancer (PTC)

## Abstract

**Purpose:**

To develop and validate a three-dimensional ultrasound (3D US) radiomics nomogram for the preoperative prediction of extrathyroidal extension (ETE) in papillary thyroid cancer (PTC).

**Methods:**

This retrospective study included 168 patients with surgically proven PTC (non-ETE, n = 90; ETE, n = 78) who were divided into training (n = 117) and validation (n = 51) cohorts by a random stratified sampling strategy. The regions of interest (ROIs) were obtained manually from 3D US images. A larger number of radiomic features were automatically extracted. Finally, a nomogram was built, incorporating the radiomics scores and selected clinical predictors. Receiver operating characteristic (ROC) curves were performed to validate the capability of the nomogram on both the training and validation sets. The nomogram models were compared with conventional US models. The DeLong test was adopted to compare different ROC curves.

**Results:**

The area under the receiver operating characteristic curve (AUC) of the radiologist was 0.67 [95% confidence interval (CI), 0.580–0.757] in the training cohort and 0.62 (95% CI, 0.467–0.746) in the validation cohort. Sixteen features from 3D US images were used to build the radiomics signature. The radiomics nomogram, which incorporated the radiomics signature, tumor location, and tumor size showed good calibration and discrimination in the training cohort (AUC, 0.810; 95% CI, 0.727–0.876) and the validation cohort (AUC, 0.798; 95% CI, 0.662–0.897). The result suggested that the diagnostic efficiency of the 3D US-based radiomics nomogram was better than that of the radiologist and it had a favorable discriminate performance with a higher AUC (DeLong test: p < 0.05).

**Conclusions:**

The 3D US-based radiomics signature nomogram, a noninvasive preoperative prediction method that incorporates tumor location and tumor size, presented more advantages over radiologist-reported ETE statuses for PTC.

## Introduction

Papillary thyroid cancer (PTC) is the most common endocrine malignant tumor, and its incidence rate is increasing all over the world (1). The reason for this increase in incidence is partly because of the popularization of routine physical examination and the improvement of high-frequency ultrasound (US) (2). Most PTC patients have a good prognosis; more than 90% of them have survived for more than 10 years (3). Although PTC has a favorable prognosis, some cases show aggressive clinical features, such as lymph node and distance metastases, and poorer prognosis (4). Extrathyroidal extension (ETE) has long been considered to be an independent predictor of poor prognosis in PTC patients (5). Also, ETE is regarded to be an important risk factor associated with recurrence and metastasis, and it has an important impact on staging and the choice of operation (6). The recurrence and mortality after surgery will increase in an ETE patient. The 15-year survival rate among PTC patients with ETE was significantly worse than that in patients without ETE (7, 8). Traditional surgical setting includes total and subtotal thyroidectomies for PTC, according to the National Comprehensive Cancer Network (NCCN) Guidelines for Thyroid Carcinoma (Third Edition, 2018); total thyroidectomy is the best method to treat ETE patients in PTC (7). Although both surgical procedures have no significant effect on distant metastasis and cancer-specific mortality rates, subtotal thyroidectomies can retain some functionality of the thyroid gland and prevent injuries of the parathyroid and contralateral laryngeal recurrent nerve (9). Therefore, predicting ETE preoperatively is critical for clinicians to choose the surgical approach.

Currently, only pathological biopsy is the gold standard for the diagnosis of ETE (10). Imaging methods such as magnetic resonance imaging (MRI), computerized tomography (CT), single-photon emission computed tomography (SPECT), and US are commonly used for the diagnosis of ETE. MRI has a high resolution of soft tissue and spatial resolution. In a previous study, Wei et al. (11) used multiparametric MRI for preoperative assessment of ETE in 132 cases of PTC, with areas under the receiver operating characteristic curve (AUCs) of 0.96 and 0.87 in the training and testing sets, respectively. However, MRI is relatively expensive and time-consuming; it is also not suitable for patients with implants or who have claustrophobia (12). CT has a certain advantage in evaluating the relationships between the lesions and surrounding tissue, but CT has a potential risk of radiation exposure. From the SPECT examination, the anatomic location of the tumor can be shown clearly. Moreover, a study suggested that SPECT also exhibits promising advantages including higher sensitivity (50%) and specificity (100%) (13). Although several studies have suggested the value of the nuclear medicine method, it could not be widely used clinically in thyroid carcinoma for reasons of universality, radiation, and cost-effectiveness. At present, US examination has become the most frequently used imaging method in PTC patients because of a series of advantages, such as being inexpensive, being noninvasive, and using non-ionizing radiation. Kwak et al. (14) reported that the sensitivity of US examination was 65.2% and the specificity was 81.8% when more than 25% of thyroid nodules contacts with the adjacent capsule. For >50% contact between the tumor and capsule, Lee et al. (15) reported that US findings of capsule disruption had a better AUC (0.674 vs. 0.638) in predicting ETE than CT in 377 PTC patients, while CT combined with US imaging to detect ETE could get the best diagnostic accuracy, with an AUC of 0.744.

With the development of US technology, the emergence of three-dimensional (3D) US provides more possibilities in choosing the imaging method of thyroid disease. Through scanning the target organs by a single sweep of a US beam, it can easily provide the images in multiple slices and planes from the stored data. Therefore, 3D imaging can provide significantly more information about lesions than the traditional two-dimensional (2D) imaging. According to several previous research reports, 3D US examination may help to overcome the limitations of 2D US in various organs (16, 17). Kim et al. (18) reported that compared to 2D thyroid US, 3D had higher sensitivity for predicting ETE (66.7% vs. 46.4%, p = 0.03). Consequently, 3D US may have diagnostic potential in predicting ETE status in PTC patients.

Radiomics is an emerging and burgeoning subject in medical research, especially in oncology. Radiomics analysis was first reported by Lambin et al. (19) in 2012. By extracting and analyzing a large number of quantitative features from medical images, radiomics can improve the ability of disease diagnosis and prediction (20, 21). Studies have shown that image feature-based radiomics extraction has objective characteristics and great value in predicting clinical outcomes (19). Radiomics analysis has been applied in various diseases, such as cervical cancer, breast cancer, prostate cancer, lung cancer, rectum cancer, and musculoskeletal tumors (22–27). In the thyroid grand, Wang et al. (28) believe that 2D US radiomics can effectively evaluate whether ETE occurs in papillary thyroid carcinoma, and the AUC is 0.824. But at present, there are no reports applying 3D US radiomics to evaluate ETE in PTC.

Therefore, the purpose of this study is to construct a 3D US radiomics nomogram to predict ETE to help the doctors select the most appropriate strategy of treatment in PTC patients.

## Materials and methods

### Patient data

This retrospective study was approved by the institutional review board of our institution. Informed consent was obtained from participants before the examination. Between November 2020 and October 2021, a total of 168 individuals who underwent preoperative 2D and 3D thyroid US at our institute were included in this study. All patients underwent subtotal or total thyroidectomy within 1 week after US examination. PTC was confirmed by pathology postoperatively. Exclusion criteria were as follows: 1) the clinical information of the patients was incomplete; 2) the patients had been submitted to thyroidectomy; 3) the entire lesion was not covered by the scan; 4) the images had poor quality; 5) the patients only had routine preoperative 2D or 3D US examination; 6) the maximum diameter of the primary tumor was <5 mm.

#### US examination and US-reported ETE status

**Figure 1:**
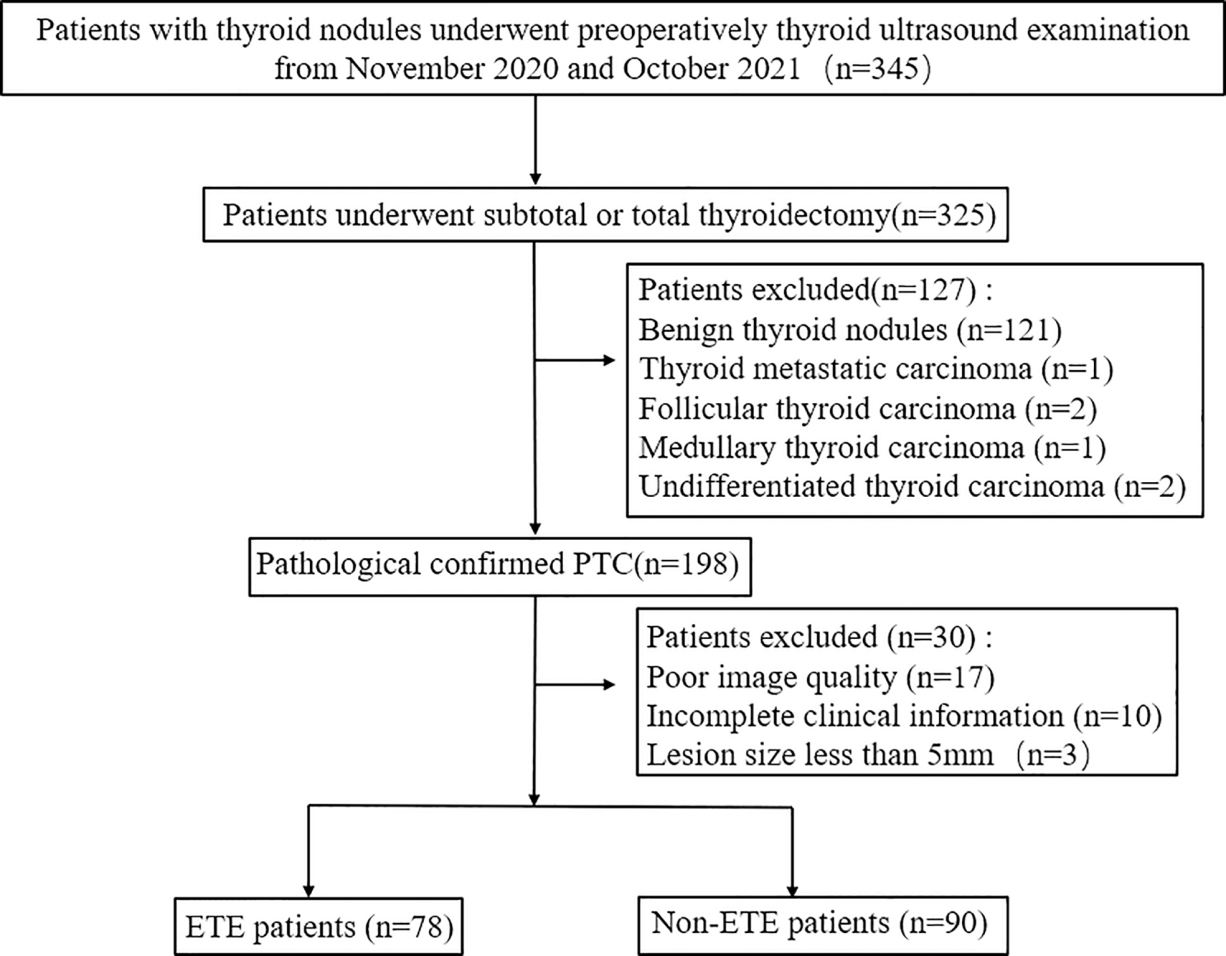
Flowchart of patient selection in this study. PTC, papillary thyroid cancer; ETE, extrathyroidal extension.

**Figure 1** shows the study procedure. Before receiving total and subtotal thyroidectomies, all patients underwent 2D US with 12-5 MHz linear array transducer and 3D US with 13-5 MHz dedicated volume transducer (Philips IU Elite Ultrasound System). The patients were placed in a supine position appropriately with a pillow underneath the neck, with their neck stretched sufficiently to expose the anterior region of the neck. All images were obtained at identical instrument settings for depth, focus position, and gain setting. Clinical data, such as age, sex, and body mass index (BMI), were obtained during the US examination.

After completing the 2D thyroid examination, all subjects underwent 3D examination by two experienced radiologists who were blinded to the data sample identity (8 and 10 years of experience in thyroid US). For 3D US, a volume box size was chosen to cover the tumor lesion. The sectorial and mechanical transducer with a scanning angle of 30° was used for automatic acquisition of tilt-series and 3D images. The two radiologists received a training course consisting of 20 unregistered cases to familiarize themselves with 3D scanning before this study. During the 2D and 3D US examination, they recorded the size of the lesion, nodule position (upper pole, middle pole, or lower pole), primary site (left lobe, right lobe, or isthmus), nodule border (clear or fuzzy), internal echo pattern (nonuniform or uniform), and nodule location (unilateral or bilateral). And they independently reviewed the US imaging features of every patient and recorded a final diagnosis. In case of disagreement, additional reading sessions were used until a consensus was reached. Based on the American Joint Committee on Cancer (AJCC) guidelines (29–32), ETE can be diagnosed when one of the following criteria presents: 1) >25% of the primary tumor perimeter is in contact with the thyroid capsule; 2) the glands between the lesions and thyroid disappear; 3) the primary tumor exceeds the thyroid capsule and extends to the surrounding structures, such as the larynx, recurrent laryngeal nerve, trachea, vasculature, the strap muscles, or esophagus.

### Tumor segmentation and radiomic feature extraction

**Figure 2:**
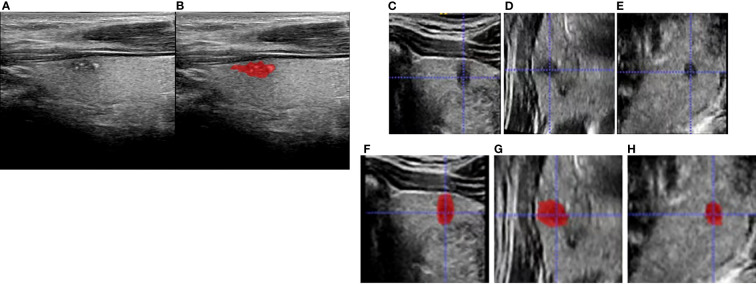
Schematic diagram for region of interest (ROI) delineation of ultrasound image: **(A)** was the original two-dimensional image during ultrasound (US) examination; **(B)** was the ROI delineated in the largest section of the tumor based on the original two-dimensional ultrasound image. **(C–E)** are transverse, coronal, and sagittal planes, respectively, and **(F–H)** are corresponding three-dimensional ROIs.

The two abovementioned radiologists were informed about the tumor location confirmed by operation and were blinded to other pathologic results and clinical information. The regions of interest (ROIs) of the 3D images were drawn layer by layer manually by the radiologists using the software ITK-SNAP (version 3.8.0, http://www.itksnap.org). Lastly, all final tumor regions could be defined by the overlapping region of two ROIs independently drawn by the two radiologists. In case of disagreement, additional reading sessions were used until a consensus was reached. **Figure 2** shows a typical case with 2D and 3D US images and the ROIs. Texture analysis was performed on the acquired US images of 168 patients. All feature extraction methods were performed using PyRadiomics package, which was imported from the Python programming language. Subsequently, a total of 1,693 features were extracted for each patient, including First Order Statistics (19 features), Shape-based (3D) (16 features), Shape-based (2D) (10 features), Gray-Level Co-Occurrence Matrix (24 features), Gray-Level Run Length Matrix (16 features), Gray-Level Size Zone Matrix (16 features), Neighboring Gray Tone Difference Matrix (5 features), and Gray-Level Features Matrix (14 features).

### Feature selection and radiomics signature building

All data were divided into training and validation sets at a ratio of 7:3 according to random stratified sampling strategy. First, Levene tests were performed to verify variance homogeneity. For two groups, we used the independent-sample t-test or Mann–Whitney U test to acquire significant features with p-values <0.05. After that, the least absolute shrinkage and selection operator (LASSO) regression method with 10-fold cross-validation was applied to select the most useful predictive ETE status-related features from the training cohort. The LASSO is a machine learning regression analysis technique; it not only can reduce model overfitting and improve the results of prediction but also is regarded as a promising method to select significant features through regularization and variable selection (33). A formula was generated using a linear combination of selected features that were weighted by their respective LASSO coefficients, and the formula was then used to calculate a risk score (defined as the radiomics score or radiomics signature). The radiomics signature was then used to build a nomogram combining with clinical predictors.

### Development of the ultrasound radiomics nomogram

The chi-square test (categorical variables) and Student’s t-test (continuous variables) were adopted to identify the association between the clinical risk factors and ETE. In this study, clinical predictors including age, sex, BMI, lesion, nodule location, nodule position, nodule border, internal echo pattern, and radiological ETE diagnosis were used to conduct a multivariate logistic regression model. To find the incremental value of the radiomics signature for prediction of ETE in PTCs, the radiomics model was built by combining the radiomics signature with clinical predictors with p < 0.05. Next, the model was converted into a radiomics nomogram to help clinicians predict ETE visually in PTC patients. We calculated the AUC for predictive analysis. Then, the DeLong test was performed to compare the differences between the receiver operating characteristic (ROC) curves of the nomogram and the radiologists.

### Assessment of nomogram performance

The 3D US-based radiomics nomogram incorporating selected clinical predictors was developed on the training cohort and then tested in the validation cohort. The calibration curve and Hosmer–Lemeshow (H-L) test were used to assess the calibration of the radiomics nomogram. The discriminative performance of the radiomics nomogram was evaluated by using Harrell’s concordance index (C-index).

#### Histopathologic analysis

Two experienced pathologists with 9 and 12 years of experience, respectively, evaluated the histopathology of the tumor specimens. PTC specimens of paraffin embedding slice were followed by hematoxylin and eosin (H&E) dyeing. According to the guidelines published by the American Thyroid Association (ATA), the pathologists evaluated the ETE features (34). Then, the patients were divided into two groups: ETE and non-ETE groups.

#### Statistical analysis

All statistical tests in this study were conducted using R software (version 4.0.3, https://www.r-project.org). Statistical analysis of clinical data, multivariate logistic regression, and H-L test were performed using SPSS software (version 22.0, SPSS Inc.). The corresponding 95% confidence interval (CI) was used to describe the correlation results. If the measurement data satisfy normal distribution, we use mean ± standard deviation (SD) to express. Other values were reported as median and interquartile range (IQR). Independent-sample t-test was adopted for normally distributed measurement data; otherwise, Mann–Whitney U test was used for non-normally distributed measurement data. The count dates were expressed as frequency (percentage) and compared by chi-square test or Fisher exact test. The factors of ETE in PTC patients were analyzed by multivariate logistic regression (stepwise forward) method. Next, the goodness of fit for logistic regression models was assessed by the H-L test.

ROC was employed to quantify the discriminative capability of the nomogram by comparing nomogram-predicted versus the observed ETE probability. A two-sided p < 0.05 was considered statistically significant.

## Results

### Clinical characteristics

A total of 168 PTC patients aged 41.96 ± 0.881 years (range, 20–72 years) were enrolled in this research. In this study, 90 patients (41.71 ± 12.73 years old) and 78 patients (42.24 ± 11.22 years old) were assigned to the non-ETE and ETE groups, respectively, based on pathologic results. There was no significant difference in the tumor size, nodule location, nodule position, nodule border, and internal echo pattern between the ETE group and non-ETE group (all p > 0.05). According to the degree of the diagnostic criteria of ETE, there were 10 patients with thyroid capsule contact approximately >25% of the primary tumor perimeter, 47 patients with the glands between the lesions and thyroid disappear, 21 patients with the primary tumor that exceeds the thyroid capsule and extends to surrounding structures. The 168 patients were divided into a training group (n = 117) and a validation group (n = 51) by stratified sampling. **Table 1** shows the clinical data of the 168 patients.

**Table 1:** Basic clinical data for our group.

	Training Set(n=117)			Validation Set(n=51)		
Variable	ETE(n=54)	Non-ETE (n=63)	p Value	ETE(n=24)	Non-ETE (n=27)	p Value
Age mean ± SD,years	42.50 ± 11.18	40.89 ± 11.61	0.634	42.33 ± 11.22	42.67 ± 11.34	0.914
Sex						
Women	28(51.85%)	42(66.67%)	0.103	17(70.83%)	21(77.78%)	0.570
Men	26(48.15%)	21(33.33%)		7(29.17%)	6(22.22%)	
BMI mean ± SD,kg/m^2^	23.45 ± 3.92	22.79 ± 3.73	0.360	22.97 ± 3.24	22.67 ± 3.82	0.765
Radiomics score mean	0.48 ± 0.02	0.51 ± 0.02	<0.001	0.50 ± 0.01	0.50 ± 0.01	<0.001
Tumor size(cm)						
≥1	31(57.41%)	16(25.40%)	<0.001	20(83.33%)	10(37.04%)	0.001
<1	23(42.59%)	47(74.60%)		4(16.67%)	17(62.96%)	
Tumor border						
Clear	21(38.89%)	24(38.10%)	0.930	11(45.83%)	12(44.44%)	0.921
Fuzzy	33(61.11%)	39(61.90%)		13(54.17%)	15(55.56%)	
Tumor location						
Unilateral	14(25.93%)	31(49.21%)	0.010	5(20.83%)	14(51.85%)	0.020
Bilateral	40(74.07%)	32(50.79%)		19(79.17%)	13(48.15%)	
Internal echo pattern						
Uniform	21(38.89%)	30(47.62%)	0.342	12(50.00%)	12(44.44%)	0.692
Nonuniform	33(61.11%)	33(52.38%)		12(50.00%)	15(55.56%)	
Radiological ETE diagnosis						
ETE	25(46.30%)	16(25.40%)	0.018	5(20.83%)	13(25.49%)	0.042
Non- ETE	29(53.70%)	47(74.60)		19(79.17%)	14(27.45%)	
Tumor position						
Upper pole	12(22.22%)	9(14.29%)	0.148	5(20.83%)	2(7.41%)	0.104
Middle pole	22(40.74%)	37(58.73%)		10(41.67%)	19(70.37%)	
Inferior pole	20(37.04%)	17(26.98%)		9(37.50%)	6(22.22%)	
Primary site						
Left lobe	27(50.00%)	24(38.10%)	0.198	9(37.50%)	12(44.44%)	0.524
Right lobe	26(48.15%)	34(53.97%)		14(58.33%)	15(55.56%)	
Isthmus	1(1.85%)	5(7.93%)		1(41.67)	0	

ETE, extrathyroidal extension; non-ETE, without extrathyroidal extension; SD, standard deviation.

### Radiomics score

**Figure 3:**
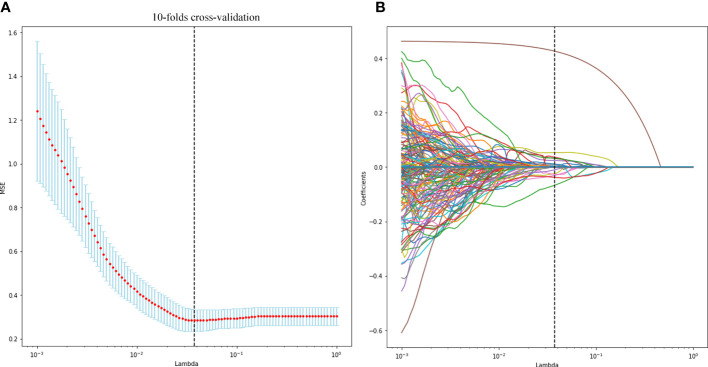
Radiomic feature selection using the least absolute shrinkage and selection operator (LASSO) regression model. The LASSO regression with 10-fold cross-validation **(A)** was used to reduce the dimension of the grouping characteristics **(B)**. Finally, 16 radiomic features with non-zero coefficients were selected.

A total of 1,693 features were extracted from the original 3D US images in the training cohort. In this study, LASSO regression with L1 regularization was further used to select the optimal radiomic features. The complexity depends on the lambda (λ). According to 10-fold cross-validation, the results indicated that when extracting 3D image features, the models had the lowest mean squared error (MSE) when λ was 0.037. After LASSO regression analysis, 691 radiomic features were reduced to 16 potential predictors. As **Figure 3** shows, the 16 features were included in the radiomics score formula. **Table 2** shows the 16 best radiomic features in 3D US image.

**Table 2:** The 16 best radiomic features in 3D US image.

Feature variable	Coefficient
original_gldm_DependenceNonUniformityNormalized	-0.067
gradient_glrlm_ShortRunEmphasis	0.031
l lbp-2D_firstorder_InterquartileRange	-0.031
squareroot_glrlm_ShortRunLowGrayLevelEmphasis	-0.024
wavelet-LHL_firstorder_Kurtosis	0.001
wavelet-LHH_firstorder_Entropy	0.008
wavelet-LHH_firstorder_Maximum	0.054
wavelet-LHH_gldm_DependenceEntropy	-0.027
wavelet-HLL_glszm_SmallAreaHighGrayLevelEmphasis	0.024
wavelet-HLH_glcm_JointEnergy	-0.010
wavelet-HLH_glrlm_LongRunHighGrayLevelEmphasis	0.037
wavelet-HHH_glcm_MaximumProbability	0.037
wavelet-HHH_gldm_HighGrayLevelEmphasis	-0.038
wavelet-HHH_glrlm_HighGrayLevelRunEmphasis	0.003
wavelet-HHH_glrlm_LowGrayLevelRunEmphasis	-5.049
wavelet-LLL_glcm_ClusterShade	0.033

### Development and performance of the prediction model

**Figure 4:**
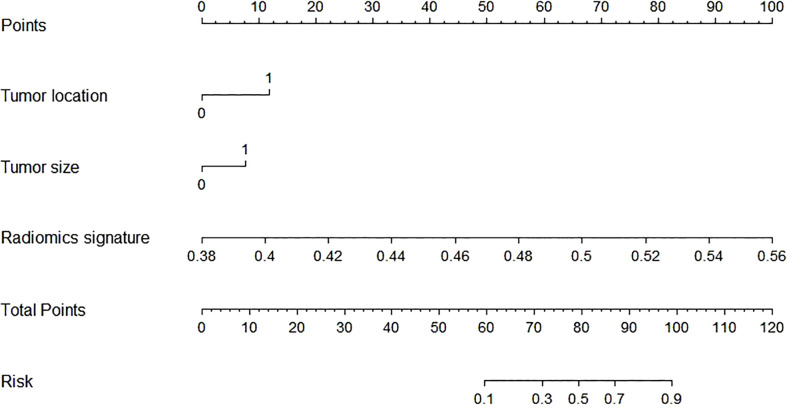
The radiomics nomogram was developed by incorporating the radiomics scores and selected clinical predictors. The radiomics nomogram incorporated tumor location (unilateral or bilateral), tumor size, and the radiomics signature.

In this study, the AUC of the radiologists is 0.67 (95% CI, 0.580–0.757) in the training cohort and 0.62 (95% CI, 0.467–0.746) in the validation cohort. Then, according to the forward Logistic regression (LR) method, bilateral tumor (p = 0.004)and tumor size (p = 0.005) were identified as independent predictive factors to predictive ETE. Then, we could get a radiomics nomogram with the predictive factors (**Figure 4**). The prediction model was constructed as follows:

Linear predictor = -27.722 - 1.153 × X1 -1.148 × X2 + 57.033 × X3. X1 being tumor location, X2 being tumor size, X3 being radiomics signature.

**Figure 5:**
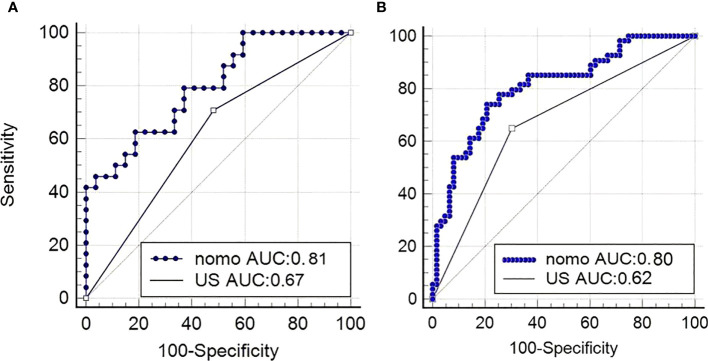
Performance of the nomogram. **(A)** ROC curves of US-reported ETE status and radiomics nomogram for predicting ETE in the validation cohort and in the primary cohort **(B)**. nomo, nomogram; US, ultrasound; ROC, receiver operating characteristic; ETE, extrathyroidal extension.

**Figure 6:**
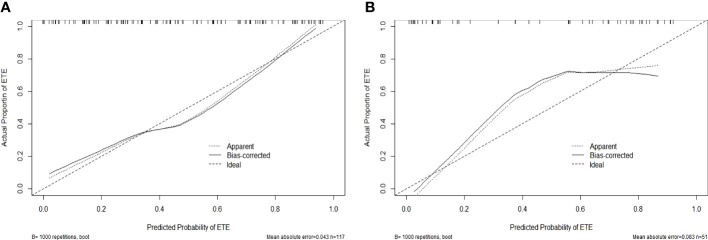
Calibration curves for the radiomics nomogram in the training **(A)** and validation **(B)** sets. The calibration curve and the Hosmer–Lemeshow test showed good calibration in the training cohort (p = 0.828) and in the validation cohort (p = 0.071).

In the training cohort, the radiomics nomogram showed good discrimination with an AUC 0.810 (95% CI, 0.727–0.876), which was significantly higher than that of the radiologists (DeLong test, p = 0.0136). In the validation set, it also shows better discrimination with an AUC of 0.798 (95% CI, 0.662–0.897; DeLong test, p = 0.0296). The ROC curves of the two models for both the training and validation sets are presented in **Figure 5**. The calibration curve and the H-L test showed good calibration in the training cohort (**Figure 6A**, p = 0.828) and the validation cohort (**Figure 6B**, p = 0.071). The C-index of the radiomics nomogram is 0.831. From the result, we could know that the above-described radiomics nomogram performed well in differentiating ETE from non-ETE and may help in the clinical decision-making process.

## Discussion

Due to the prevalence of coronavirus disease 2019 (COVID-19), delayed investigations and treatment may further lead to an increase in the incidence and mortality of thyroid carcinoma (35). As the most common pathological type of differentiated thyroid carcinoma (DTC), PTC has a low degree of malignancy and a high cure rate if detected and treated in a timely manner. The presence of ETE is considered to be significantly important in PTC patients, and it is included in almost all prognostic scoring systems as a staging variable. Literature reported that the incidence of ETE in PTC ranges between 5% and 45%, and in our patient population, the incidence of ETE was 46% (78/168); the values did not differ much (36). Moreover, PTC patients with ETE have poor prognosis and a high risk of recurrence in the near future (37). And a study found that patients with a history of ETE display a poorer therapeutic effect following 131I ablation (38). The thyroid capsule is closely adjacent to the surrounding tissues such as recurrent laryngeal nerve, the patients who are not diagnosed correctly before surgery often need reoperation, which increases the pain and economic burden of PTC patients. Taken together, it is very important to identify the presence of ETE before thyroid surgery.

At present, only pathological diagnosis is the gold standard of ETE diagnosis. Reliable and accurate preoperative imaging examination of ETE will help in accurate diagnosis and selection of the optimum therapeutic strategy. Although fine-needle aspiration biopsy (FNAB) is the most widely used method for the assessment of thyroid nodules preoperatively, it is an invasive procedure, and its samples cannot always reach satisfying diagnostic power. Alternatively, the diagnostic capacity for ETE in PTC patients is limited (39). Noninvasive imaging examinations such as ultrasonic imaging, MRI, CT, and positron emission tomography not only play an increasingly important role in the diagnosis of ETE but also can be useful to guide treatment and patient follow-up. Among them, US is the sole fundamental imaging modality for the assessment of thyroid nodules. US examination is noninvasive, fast, and reliable and can help to enhance the early detection of pathologies (40–42). However, these traditional imaging examinations depend on the experience and subjective judgments of the radiologists, which limit the ability to make an objective decision.

Therefore, a noninvasive sample and quantitative methods are needed to help clinically predict ETE. Radiomics is a hot topic; it allows the quantitative extraction of high-throughput features from radiographic images, with the advantage of objectivity. Numerous studies have confirmed the usefulness of 3D US in clinical settings. Based on the transverse and longitudinal views of 2D US, 3D US technology improves the visualization of target lesions by adding a new dimension, coronal view. In a study involving 85 PTC patients, Kim et al. (18) believed that 3D US not only had higher sensitivity (66.7% vs. 46.4%, p = 0.03) but also showed better agreement (k 0.53 vs. 0.37) than 2D for predicting ETE, and 3D thyroid US saved time for scanning compared with 2D. But at present, there are no reports applying 3D US radiomics to compare the diagnostic performance to evaluate ETE in PTC, which is the main novelty of this research paper.

Kim et al. (43) found that the tumor size ≥1 cm was considered to be a dependent prognostic factor to predict ETE in PTC. In our study, after forward LR method, we found that the PTC patients with tumor size ≥1 cm had been associated with ETEs, which was in line with the above findings. We also found that the location of the nodules (unilateral or bilateral) was another clinical predictor. Then, we built a radiomics nomogram based on the clinical risk factors, tumor size and tumor location, to predict ETE. As the result showed, the AUC of the radiomics nomogram in diagnosing ETE was higher than that of the radiologists in both the training cohort and the validation cohort. In addition, the C-index and calibration curve also showed good consistency in the primary group and validation group. Hence, the 3D US-based radiomics signature nomogram, a noninvasive preoperative prediction method that incorporates tumor location and tumor size, presented more advantages over radiologist-reported ETE statuses for PTC, which could be a visualization tool for the clinic to choose a suitable surgical modality.

Inevitably, our present study has several limitations. First, this study was a retrospective and single-center study, lacking external validation. Therefore, these results are not necessarily suitable to all patients with PTC preoperatory; multicenter studies are warranted to further validate the results. Moreover, to improve the diagnostic capability, the algorithm and models will be further optimized to improve the accuracy of external validation. Second, in this study, the radiomic features might not be sufficient because we only used grayscale US images to perform the radiomics nomogram. In future research, we will add radiomic characteristics of multimodal US such as elastography and contrast-enhanced US images to the nomogram. Finally, the ROI segmentation was obtained manually, which might be affected by the radiologist’s subjective bias. And it may be resolved by semiautomatic/automatic segmentation.

Our 3D US-based radiomics nomogram combining clinical predictors, tumor location and tumor size, shows favorable predictive accuracy for preoperative ETE in patients with PTC. This nomogram is a promising tool to improve the diagnostic accuracy.

## Data availability statement

The original contributions presented in the study are included in the article/supplementary material. Further inquiries can be directed to the corresponding authors.

## Ethics statement

The studies involving human participants were reviewed and approved by The Second Affiliated Hospital of Guangzhou Medical University. The patients/participants provided their written informed consent to participate in this study. Written informed consent was obtained from the individual(s) for the publication of any potentially identifiable images or data included in this article.

## Author contributions

W-JL: investigation, data curation, formal analysis, conceptualization, writing–original draft, and writing–review and editing. LM: investigation, data curation, formal analysis, conceptualization, writing–original draft, and writing–review and editing. JL: data curation, writing–original draft, and writing–review and editing. Y-WW: data curation, writing–original draft, and writing–review and editing. S-NC: writing–original draft and writing–review and editing. Y-YL: writing–original draft and writing–review and editing. L-YO: writing–original draft and writing–review and editing. J-YC: writing–original draft and writing–review and editing. S-YC: writing–original draft and writing–review and editing. S-DQ: supervision, formal analysis, conceptualization, writing–original draft, and writing–review and editing. FC: supervision, formal analysis, conceptualization, writing–original draft, and writing–review and editing. All authors contributed to the article and approved the submitted version.

## References

[1] Lim H Devesa SS Sosa JA Check D Kitahara CM. Trends in thyroid cancer incidence and mortality in the United States, 1974-2013. JAMA (2017) 317:1338–48. doi: 10.1001/jama.2017.2719 PMC8216772 28362912

[2] Yu J Deng Y Liu T Zhou J Jia X Xiao T. Lymph node metastasis prediction of papillary thyroid carcinoma based on transfer learning radiomics. Nat Commun (2020) 11:4807. doi: 10.1038/s41467-020-18497-3 32968067 PMC7511309

[3] Pamedytyte D Simanaviciene V Dauksiene D Leipute E Zvirbliene A Sarauskas V. Association of MicroRNA expression and BRAF(V600E) mutation with recurrence of thyroid cancer. Biomolecules (2020) 12(7):10. doi: 10.3390/biom10040625 PMC7226510 32316638

[4] Chen F Jin Y Feng L Zhang J Tai J Shi J. RRS1 gene expression involved in the progression of papillary thyroid carcinoma. Cancer Cell Int (2018) 18:20. doi: 10.1186/s12935-018-0519-x 29449788 PMC5812111

[5] Yao Y Chen X Yang H Chen W Qian Y Yan Z. I_circ_0058124 promotes papillary thyroid cancer tumorigenesis and invasiveness through the NOTCH3/GATAD2A axis. J Exp Clin Cancer Res (2019) 38:318. doi: 10.1186/s13046-019-1321-x 31324198 PMC6642504

[6] Kamaya A Tahvildari AM Patel BN Willmann JK Jeffrey RB Desser TS. Sonographic detection of extracapsular extension in papillary thyroid cancer. J Ultrasound Med (2015) 34:2225–30. doi: 10.7863/ultra.15.02006 26518279

[7] Shaha AR. Implications of prognostic factors and risk groups in the management of differentiated thyroid cancer. Laryngoscope (2004) 114:393–402. doi: 10.1097/00005537-200403000-00001 15091208

[8] Sundram F Robinson BG Kung A Lim-Abrahan MA Bay NQ Chuan LK. Well-differentiated epithelial thyroid cancer management in the Asia Pacific region: A report and clinical practice guideline. Thyroid (2006) 16:461–9. doi: 10.1089/thy.2006.16.461 16756468

[9] Hay ID Grant CS Bergstralh EJ Thompson GB van Heerden JA Goellner JR. Unilateral total lobectomy: Is it sufficient surgical treatment for patients with AMES low-risk papillary thyroid carcinoma? Surgery (1998) 124:958–64. doi: 10.1016/S0039-6060(98)70035-2 9854569

[10] Miller B Burkey S Lindberg G Snyder WR Nwariaku FE. Prevalence of Malignancy within cytologically indeterminate thyroid nodules. Am J Surg (2004) 188:459–62. doi: 10.1016/j.amjsurg.2004.07.006 15546550

[11] Wei R Wang H Wang L Hu W Sun X Dai Z. Radiomics based on multiparametric MRI for extrathyroidal extension feature prediction in papillary thyroid cancer. BMC Med Imaging (2021) 21:20. doi: 10.1186/s12880-021-00553-z 33563233 PMC7871407

[12] Tang CY Wang VX Lun MY Mincer JS Ng JC Brallier JW. Transient changes in white matter microstructure during general anesthesia. PloS One (2021) 16:e247678. doi: 10.1371/journal.pone.0247678 PMC7997710 33770816

[13] Barwick T Murray I Megadmi H Drake WM Plowman PN Akker SA. Single photon emission computed tomography (SPECT)/computed tomography using Iodine-123 in patients with differentiated thyroid cancer: Additional value over whole body planar imaging and SPECT. Eur J Endocrinol (2010) 162:1131–9. doi: 10.1530/EJE-09-1023 20212015

[14] Kwak JY Kim EK Youk JH Kim MJ Son EJ Choi SH. Extrathyroid extension of well-differentiated papillary thyroid microcarcinoma on US. Thyroid (2008) 18:609–14. doi: 10.1089/thy.2007.0345 18578609

[15] Lee DY Kwon TK Sung MW Kim KH Hah JH. Prediction of extrathyroidal extension using ultrasonography and computed tomography. Int J Endocrinol (2014) 2014:351058. doi: 10.1155/2014/351058 25525431 PMC4265702

[16] Cho N Moon WK Cha JH Kim SM Han BK Kim EK. Differentiating benign from Malignant solid breast masses: Comparison of two-dimensional and three-dimensional US. Radiology (2006) 240:26–32. doi: 10.1148/radiol.2401050743 16684920

[17] Li QY Tang J He EH Li YM Zhou Y Zhang X. Clinical utility of three-dimensional contrast-enhanced ultrasound in the differentiation between noninvasive and invasive neoplasms of urinary bladder. Eur J Radiol (2012) 81:2936–42. doi: 10.1016/j.ejrad.2011.12.024 22260895

[18] Kim SC Kim JH Choi SH Yun TJ Wi JY Kim SA. Off-site evaluation of three-dimensional ultrasound for the diagnosis of thyroid nodules: Comparison with two-dimensional ultrasound. Eur Radiol (2016) 26:3353–60. doi: 10.1007/s00330-015-4193-2 26795614

[19] Lambin P Rios-Velazquez E Leijenaar R Carvalho S van Stiphout RG Granton P. Radiomics: Extracting more information from medical images using advanced feature analysis. Eur J Cancer (2012) 48:441–6. doi: 10.1016/j.ejca.2011.11.036 PMC4533986 22257792

[20] Gillies RJ Kinahan PE Hricak H. Radiomics: Images are more than pictures, they are data. Radiology (2016) 278:563–77. doi: 10.1148/radiol.2015151169 PMC4734157 26579733

[21] Yip SS Aerts HJ. Applications and limitations of radiomics. Phys Med Biol (2016) 61:R150–66. doi: 10.1088/0031-9155/61/13/R150 PMC4927328 27269645

[22] Park SH Lim H Bae BK Hahm MH Chong GO Jeong SY. Robustness of magnetic resonance radiomic features to pixel size resampling and interpolation in patients with cervical cancer. Cancer Imaging (2021) 21:19. doi: 10.1186/s40644-021-00388-5 33531073 PMC7856733

[23] Conti A Duggento A Indovina I Guerrisi M Toschi N. Radiomics in breast cancer classification and prediction. Semin Cancer Biol (2021) 72:238–50. doi: 10.1016/j.semcancer.2020.04.002 32371013

[24] Avanzo M Stancanello J Pirrone G Sartor G. Radiomics and deep learning in lung cancer. Strahlenther Onkol (2020) 196:879–87. doi: 10.1007/s00066-020-01625-9 32367456

[25] Huang YQ Liang CH He L Tian J Liang CS Chen X. Development and validation of a radiomics nomogram for preoperative prediction of lymph node metastasis in colorectal cancer. J Clin Oncol (2016) 34:2157–64. doi: 10.1200/JCO.2015.65.9128 27138577

[26] Chianca V Albano D Messina C Vincenzo G Rizzo S Del GF. An update in musculoskeletal tumors: From quantitative imaging to radiomics. Radiol Med (2021) 126(8)1095–105. doi: 10.1007/s11547-021-01368-2 34009541

[27] Woźnicki P Westhoff N Huber T Riffel P Froelich MF Gresser E. Multiparametric MRI for prostate cancer characterization: Combined use of radiomics model with PI-RADS and clinical parameters. Cancers (Basel) (2020) 18(1):145. doi: 10.3390/cancers12071767 PMC7407326 32630787

[28] Wang X Agyekum EA Ren Y Zhang J Zhang Q Sun H. A radiomic nomogram for the Ultrasound-Based evaluation of extrathyroidal extension in papillary thyroid carcinoma. Front Oncol (2021) 11:625646. doi: 10.3389/fonc.2021.625646 33747941 PMC7970696

[29] Hay ID Johnson TR Thompson GB Sebo TJ Reinalda MS. Minimal extrathyroid extension in papillary thyroid carcinoma does not result in increased rates of either cause-specific mortality or postoperative tumor recurrence. Surgery (2016) 159:11–9. doi: 10.1016/j.surg.2015.05.046 26514317

[30] Chen W Zheng R Baade PD Zhang S Zeng H Bray F. Cancer statistics in China, 2015. CA Cancer J Clin (2016) 66:115–32. doi: 10.3322/caac.21338 26808342

[31] Cooper DS Doherty GM Haugen BR Kloos RT Lee SL Mandel SJ. Revised American Thyroid Association management guidelines for patients with thyroid nodules and differentiated thyroid cancer. Thyroid (2009) 19:1167–214. doi: 10.1089/thy.2009.0110 19860577

[32] Lee CY Kim SJ Ko KR Chung KW Lee JH. Predictive factors for extrathyroidal extension of papillary thyroid carcinoma based on preoperative sonography. J Ultrasound Med (2014) 33:231–8. doi: 10.7863/ultra.33.2.231 24449725

[33] Bien J Taylor J TibshIrani R. A lasso for hierarchical interactions. Ann Stat (2013) 41:1111–41. doi: 10.1214/13-AOS1096 PMC4527358 26257447

[34] Haugen BR. 2015 American Thyroid Association Management Guidelines for Adult Patients with Thyroid Nodules and Differentiated Thyroid Cancer: What is new and what has changed? Cancer-Am Cancer Soc (2017) 123:372–81. doi: 10.1002/cncr.30360 27741354

[35] Liang W Guan W Chen R Wang W Li J Xu K. Cancer patients in SARS-CoV-2 infection: A nationwide analysis in China. Lancet Oncol (2020) 21:335–7. doi: 10.1016/S1470-2045(20)30096-6 PMC7159000 32066541

[36] Hu S Zhang H Wang X Sun Z Ge Y Li J. Can Diffusion-Weighted MR imaging be used as a tool to predict extrathyroidal extension in papillary thyroid carcinoma? Acad Radiol (2021) 28:467–74. doi: 10.1016/j.acra.2020.03.005 32303443

[37] Chreau N Buffet C Trsallet C Tissier F Golmard JL Leenhardt L. Does extracapsular extension impact the prognosis of papillary thyroid microcarcinoma? Ann Surg Oncol (2014) 21:1659–64. doi: 10.1245/s10434-013-3447-y 24394985

[38] Li C Zhang J Wang H. Predictive value of LN metastasis detected by (18)F-FDG PET/CT in patients with papillary thyroid cancer receiving iodine-131 radiotherapy. Oncol Lett (2019) 18:1641–8. doi: 10.3892/ol.2019.10500 PMC6607093 31423231

[39] Lu Y Moreira AL Hatzoglou V Stambuk HE Gonen M Mazaheri Y. Using diffusion-weighted MRI to predict aggressive histological features in papillary thyroid carcinoma: A novel tool for pre-operative risk stratification in thyroid cancer. Thyroid (2015) 25:672–80. doi: 10.1089/thy.2014.0419 PMC4490628 25809949

[40] Intenzo CM Dam HQ Manzone TA Kim SM. Imaging of the thyroid in benign and Malignant disease. Semin Nucl Med (2012) 42:49–61. doi: 10.1053/j.semnuclmed.2011.07.004 22117813

[41] Wang J He X Ma L Li M Sun L Jiang J. Multimode ultrasonic technique is recommended for the differential diagnosis of thyroid cancer. Peerj (2020) 8:e9112. doi: 10.7717/peerj.9112 32411540 PMC7204870

[42] Merz E. Three-dimensional transvaginal ultrasound in gynecological diagnosis. Ultrasound Obstet Gynecol (1999) 14:81–6. doi: 10.1046/j.1469-0705.1999.14020081.x 10492865

[43] Kim SS Lee BJ Lee JC Kim SJ Lee SH Jeon YK. Preoperative ultrasonographic tumor characteristics as a predictive factor of tumor stage in papillary thyroid carcinoma. Head Neck (2011) 33:1719–26. doi: 10.1002/hed.21658 22076977

